# Dual Weakly Supervised Anomaly Detection and Unsupervised Segmentation for Real-Time Railway Perimeter Intrusion Monitoring

**DOI:** 10.3390/s25206344

**Published:** 2025-10-14

**Authors:** Donghua Wu, Yi Tian, Fangqing Gao, Xiukun Wei, Changfan Wang

**Affiliations:** 1 State Key Laboratory of High-Speed Maglev Transportation Technology, CRRC Qingdao Sifang Co., Ltd., Qingdao 266111, China; sf-wudonghua@cqsf.com (D.W.); tianyi@cqsf.com (Y.T.); wangchangfan@cqsf.com (C.W.); 2 School of Traffic and Transportation, Beijing Jiaotong University, Beijing 100044, China; 21120980@bjtu.edu.cn

**Keywords:** foreign object intrusion, high-speed railway, anomaly detection, foreground segmentation, weakly supervised

## Abstract

The high operational velocities of high-speed trains present constraints on their onboard track intrusion detection systems for real-time capture and analysis, encompassing limited computational resources and motion image blurring. This emphasizes the critical necessity of track perimeter intrusion monitoring systems. Consequently, an intelligent monitoring system employing trackside cameras is constructed, integrating weakly supervised video anomaly detection and unsupervised foreground segmentation, which offers a solution for monitoring foreign objects on high-speed train tracks. To address the challenges of complex dataset annotation and unidentified target detection, weakly supervised learning detection is proposed to track foreign object intrusions based on video. The pretraining of Xception3D and the integration of multiple attention mechanisms have markedly enhanced the feature extraction capabilities. The Top-K sample selection alongside the amplitude score/feature loss function effectively discriminates abnormal from normal samples, incorporating time-smoothing constraints to ensure detection consistency across consecutive frames. Once abnormal video frames are identified, a multiscale variational autoencoder is proposed for the positioning of foreign objects. A downsampling/upsampling module is optimized to increase feature extraction efficiency. The pixel-level background weight distribution loss function is engineered to jointly balance background authenticity and noise resistance. Ultimately, the experimental results indicate that the video anomaly detection model achieved an AUC of 0.99 on the track anomaly detection dataset and processes 2 s video segments in 0.41 s. The proposed foreground segmentation algorithm achieved an F1 score of 0.9030 in the track anomaly dataset and 0.8375 on CDnet2014, with 91 Frames per Second, confirming its efficacy.

## 1. Introduction

In 2019, China clearly stated that by 2035, a strong transportation country will be built, achieving the goal of covering the major cities in the country in 3 h. To achieve this goal, trains must increase operational speeds, and a more comprehensive safety monitoring system is necessary for train operations. However, high-speed rails (with speeds exceeding 200 km/h) involve multiple advanced technologies and face multiple and multi-level safety challenges [1,2,3]. Specifically, the extensive track lines span complex and dynamic environments, with challenges categorised into natural and external factors. Natural factors include extreme weather conditions such as heavy snowstorms, strong winds, and geological disasters. In addition, external threats, such as foreign object intrusions, falling debris, and high-altitude rockfalls from mountainous terrain, further add risk to the system.

To address these safety challenges, cameras can achieve real-time monitoring and rapid response to various potential safety threats. Furthermore, they have the advantages of wide coverage, uninterrupted operation in all weather conditions, and over-the-horizon detection [4] and do not need frequent personnel inspections on site. Meanwhile, due to the high speed, the on-board cameras cost more for the same image clarity and have scarcer computing resources for the operational environment than the trackside cameras. However, existing video systems generate a large amount of data. If these videos are processed through manual review and post-review methods, they will cost a large amount of labour and material costs. Nowadays, the application of computer vision has brought new solutions to this challenge [4,5]. In addition, it also has powerful data analysis capabilities, which can perform intelligent video analysis, ensuring the safe operation of the train in harsh environments.

In real life, a large amount of data obtained is not labelled. Moreover, due to the specificity of the high-speed train, it is impossible to generate or collect enough foreign object intrusion videos, leading to a data imbalance [6]. However, object detection usually requires a large amount of precisely labelled data, while weakly supervised anomaly detection [7] requires only a small number of fully labelled data, and unsupervised anomaly detection does not require fully labelled data. For the track scene, it greatly reduces the cost of detecting a large number of anomalies. The unpredictability of foreign object intrusions in track environments makes weakly supervised and unsupervised methods more suitable for railway applications due to their ability to generalise better to new anomalies without prior knowledge of all possible intrusion types. The scientific objective of this paper is to develop an intelligent vision system for real-time monitoring of the intrusion of the railway perimeter. This system aims to overcome the core challenges of data annotation costs, adaptability to complex environments, and real-time processing efficiency by integrating a dual-frame approach of weakly supervised video anomaly detection and unsupervised foreground segmentation. The main contributions of this paper are as follows.

(1) Based on the Xception3D (X3D) [8] network, a novel approach is proposed by integrating the nonlocal module [9] and multiscale temporal attention. It combines the Top-K sample selection strategy with an amplitude score/feature loss function to enhance the distinction of the classification boundary between normal and abnormal samples. At the same time, the time-smoothing constraint is incorporated to ensure the consistency of detection in continuous video frames.
(2) An improved foreground segmentation algorithm based on the Variational Autoencoder (VAE) [10] is proposed. It incorporates spatial position encoding to enhance the accuracy of target positioning. The down/upsampling modules are optimized to improve the efficiency of image feature extraction and the model size is reduced, thus accelerating the computation speed.
(3) The proposed foreground segmentation algorithm utilizes the idea of pixel-level background weight distribution and incorporates it into the design of the loss function, aiming to jointly optimize background authenticity and anti-noise performance.

The remaining sections of this paper are organised as follows. Section 2 specifically elaborates on the current methods for tracking foreign objects that invade vision, the methods for tracking foreign objects that invade video, and the current state of foreground segmentation. The weakly supervised video detection and unsupervised foreground segmentation models are thoroughly described in Section 3. Section 4 discusses the ablation experiments and comparative experiments of the model. Finally, Section 5 summarises the entire paper, and prospects are given.

## 2. Related Work

Vision-based technologies for foreign object intrusion detection encompass a diverse array of methods, which can be broadly categorised into traditional vision detection algorithms and deep learning-based detection algorithms. Traditional vision detection includes motion difference or background modelling techniques [11], weighted classification approaches that integrate scene priors and motion patterns, and methods that involve feature description and multiscale analysis [12,13]. From the perspective of motion difference, Sehchan et al. [11] develop a multicamera monitoring system for real-time observation of platform areas, using frame difference to detect intrusion by person or obstacles. However, this method is sensitive to environmental changes, leading to high false alarm rates, and threshold selection significantly affects accuracy. Regarding feature description and multiscale analysis, Thanh et al. [13] introduce a real-time pedestrian detection scheme for embedded visual systems. This system uses a histogram of orientated gradients (HOG) with selective search to reduce processing time, optimising candidate region searches via adaptive Gaussian mixture model (AGMM) background subtraction, and employing approximate HOG calculations for speed. Li et al. [12] propose an automatic intrusion detection algorithm that takes advantage of multiscale images and dynamic candidate regions. The changes in the grey projection curve are analysed to screen candidate regions and accurately extract targets from high-resolution images using background difference techniques. Comparative experiments on three videos of railway monitoring demonstrate efficiency and accuracy in real-time detection.

Track foreign object intrusion detection technologies based on deep learning can generally be divided into two categories, which are detection technologies based on 2D images [14,15,16,17] and detection technologies based on 3D vision [18,19,20,21]. In the area of detection based on 2D images, methods are classified into static image analysis [16] and video sequence analysis [22]. Static image processing employs techniques such as image segmentation [23] and object detection algorithms [24], such as Faster R-CNN and YOLO, to accurately identify and localise foreign objects on tracks. Video sequence analysis uses approaches such as optical flow and background difference. Through the analysis of continuous video frames, it effectively captures transient foreign object intrusion events that occur in the track area [25,26,27]. In 3D vision, detection focusses on point cloud processing and stereo vision. Researchers combine 3D LiDAR and other sensing devices to gather point cloud data, employing deep learning architectures such as PointNet [28] and PointCNN [29] to precisely position foreign objects in three-dimensional space. Stereo vision involves using binocular or multi-camera systems to construct depth images using parallax calculations, enhancing the identification of foreign objects through deep learning. Qi et al. [30] improve detection by preprocessing radar data to filter out interference while retaining targets within a safe range, effectively combining radar and machine vision for joint obstacle detection. Jin et al. [15] introduce a microwave radar-based method to address the limitations of machine vision in real-time performance and environmental adaptability. Their technique involves offline error correction, the construction of a clearance area in radar coordinates, and tracking moving targets, achieving a comprehensive detection rate of around 90% in complex railway environments. Although computer vision applications in track intrusion detection have made significant strides across various technologies, current research often overlooks the complexities of the data set and its impact on model generalisation. Weakly supervised and unsupervised techniques are used to streamline data set production and improve model performance in terms of real-time response, detection accuracy, and adaptability to challenging environments.

Video anomaly detection is a key technology for identifying and locating abnormal events in surveillance videos [31]. It has a wide range of applications in many fields, such as security monitoring, autonomous driving, and industrial production monitoring. The essence of tracking perimeter foreign object intrusion detection is also a video anomaly detection task. According to different data and methods, video anomaly detection [32] can be roughly divided into four categories: supervised learning, unsupervised learning, weakly supervised learning, and self-supervised learning. The following explains in detail the application status and achievements of these methods in research.

In the field of unsupervised video anomaly detection, Generative Cooperative Learning (GCL) [33] is proposed. It uses low-frequency features of abnormal events to establish cross-supervision between the generator and the discriminator, thereby enhancing its performance, all without the need for labelled data. MyeongAh et al. [34] develop the Implicit Two-Path Autoencoder (ITAE), which uses two encoders to capture visual and dynamic features. In combination with a normalised flow model, it effectively models normal feature distributions to detect anomalies. Che et al. [35] introduce an adversarial 3D convolutional autoencoder within a framework of joint learning to deep-learnt normal spatiotemporal patterns and their correlations, demonstrating superior performance in multiple public datasets.

In the field of weakly supervised video anomaly detection, Didik et al. [23] propose a method that combines the extraction of relational features, self-attention, and conditional random fields to capture spatiotemporal interactions, multiscale dependencies, and local/global relationships of CNN features in videos. Li et al. [36] design a scale-aware learning method that incorporates a spatial relationship module and multiscale patch aggregation. It effectively identifies local abnormal patterns and captures spatiotemporal dynamics, demonstrating state-of-the-art results on the UCF-Crime and ShanghaiTech dataset.

In the field of self-supervised video anomaly detection, Wu et al. [37] propose the self-supervised sparse representation framework (S3R), which combines dictionary learning and self-supervision to distinguish normal and abnormal segment features in both one-class and weakly supervised settings. Wang et al. [22] develop a spatiotemporal puzzle pretext task that decouples spatial and temporal dimensions to train models on puzzles of varying difficulty. This approach effectively captures subtle spatiotemporal differences in anomalies and outperforms traditional reconstruction-based and prediction-based methods, particularly on the ShanghaiTech dataset.

Current video anomaly detection methods primarily focus on anomalies in foreground appearance and motion patterns, which leads to insufficient attention to the contextual semantic information of the foreground. This results in a high false-positive rate when defining anomalies across various scenes and behaviours. In terms of the feature extraction of the model, more emphasis should be placed on spatiotemporal information in video. This situation highlights the importance of this paper, especially in the use of video anomaly detection methods to address intrusion detection problems of tracking.

Background extraction and foreground segmentation technologies aim to accurately distinguish objects of interest (i.e., the foreground) from relatively fixed background or non-key areas of interest in images or video [38]. In many real-life scenarios, this technology has been widely applied, covering multiple frontier fields, such as security monitoring systems, unmanned driving systems, and remote sensing of unmanned aerial vehicles [39,40,41,42]. According to differences in research methods, background extraction and foreground segmentation technologies can be divided into two categories, namely traditional methods and deep learning-based methods [43,44,45,46,47,48]. The following will further elaborate on the performance and achievements of these different methods in practical scientific research and applications.

In traditional methods, the ViBe Algorithm [49] compares the historical and current values of pixels. It adaptively updates the background model by randomly selecting pixel values and propagates updates to neighbouring pixels for robust background modelling. St-Charles et al. [50] design a new method named PAWCS, which uses a robust dictionary model based on colour and local binary characteristics and self-adjusts parameters through a feedback mechanism to adapt to complex conditions. St-Charles [51] later proposed a new universal pixel-level segmentation method called SuBSENSE. By fusing spatiotemporal binary features and colour information, it can detect concealed foreground objects more accurately while ignoring most of the illumination changes. This method uses a pixel-level feedback loop to dynamically adjust internal parameters without manual intervention.

In the field of deep learning, facing the challenges of segmentation of motion objects in complex environments, Long et al. [52] propose two architectures for segmentation of motion objects, one employing a triple encoder for multiscale information capture and the other integrating a feature pooling module for scene complexity. Both of them utilise transpose convolutions for precise segmentation in the decoding stage. Tezcan et al. [53] introduce spatiotemporal domain data enhancement techniques in response to the problem of the insufficient generalization ability of algorithms for unseen videos in the background subtraction task and applied them to the video-independent supervised background subtraction algorithm BSUV-Net, upgrading it to version 2.0. Yang et al. [54] combine multiscale spatiotemporal features using a novel layer structure to improve the detection of moving objects in video sequences. Akilan et al. [55] propose a 3D CNN-LSTM model that adopts a dual encoding and slow decoding strategy to improve the quality of the foreground representation. It improves the problem of inaccurate foreground boundaries caused by single-frame detection by making full use of spatiotemporal clues.

Although background extraction and foreground segmentation technologies have made significant progress in various scenarios, there are still some challenges and deficiencies. For example, they may show certain limitations when dealing with drastic changes in illumination, complex background dynamics, and large-scale scenes. Although there have been breakthroughs in moving object segmentation in complex environments, there are also problems, such as a large demand for training samples and the need to improve generalisation ability. The comparison results of the various studies elaborated above are shown in Table 1.

## 3. Materials and Methods

In the detection of railway video anomalies, two key issues must be addressed: one is the timing of anomalies in the video stream and the other is the category and location of these anomalies. To address the first issue, a weakly supervised video anomaly detection method is proposed. After the specific anomalous video frames are obtained, an unsupervised foreground segmentation technique is introduced to locate the anomalies within the image.

### 3.1. Weakly Supervised Video Anomaly Detection Based on the Trackside Camera

In railway environments, foreign object intrusion events are rare and diverse in types, making it costly to obtain a large number of accurately labelled abnormal samples. Weakly supervised learning only requires video-level labels to train the model, which significantly reduces the labelling burden while possessing a generalization ability for unknown abnormal types. Therefore, this paper adopts a weakly supervised video anomaly detection method.

**Figure 1:**
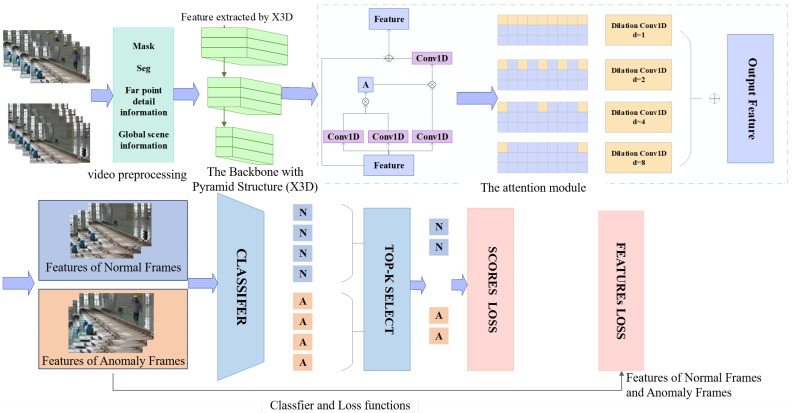
Tracking video anomaly detection based on weak supervision.

To address the issues of complex dataset annotation and unknown target detection, the framework for weakly supervised foreign object intrusion detection and tracking based on video surveillance is shown in Figure 1. It comprises the following components: video input settings; a video preprocessing module; a backbone pre-trained with large video datasets; an attention enhancement module, which includes the non-local for establishing global dependencies and the multiscale temporal attention module for establishing local temporal dependencies; a linear classifier module; Top-K selection; and a loss function encompassing the amplitude score loss function, the amplitude feature loss function, and the time-smoothing term loss function.

#### 3.1.1. Input Mask and Far-Point Information Amplification

**Figure 2:**
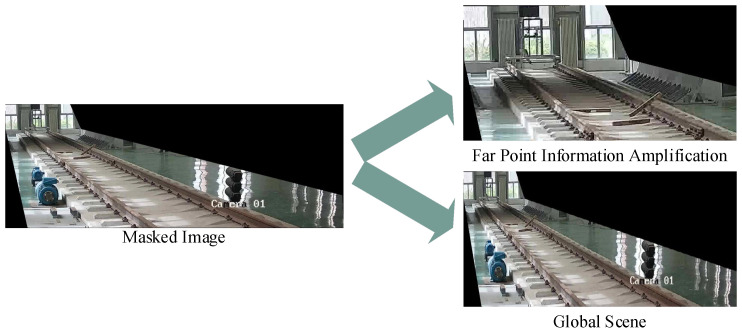
Cropping of distant image and far-point information amplification. Far-point information amplification is the image cropped from the top-left corner of the original image according to a certain size. The global scene is the image resized to the same size as the amplification of the information from the far point.

In railway scenarios, there is often a lot of interference, which requires video pre-processing to highlight key areas within the railway clearance. This pre-processing involves image mask cropping and far-point information amplification, as shown in Figure 2. Image mask cropping, based on track boundaries, uses image masks to select specific areas for regions of interest (ROIs), with uninterested areas set as background colour for easier subsequent processing. A strategy for far-point information amplification is proposed: local images in the far-point area of the track are separately cropped and combined with uncropped complete images to form a set of complementary image samples (the Far point indicates the area within the image where the target is far from the shooting position). This dual-perspective approach ensures that both macro-context and micro-details are preserved for comprehensive analysis. These samples highlight detailed features of distant targets in the current frame and reflect features of the global scene, providing additional perspective for anomaly detection and improving detection efficiency. The integration of these two image types effectively addresses the challenge of detecting small or distant objects in complex railway environments.

#### 3.1.2. Backbone Network and Attention Modules

**Figure 3:**
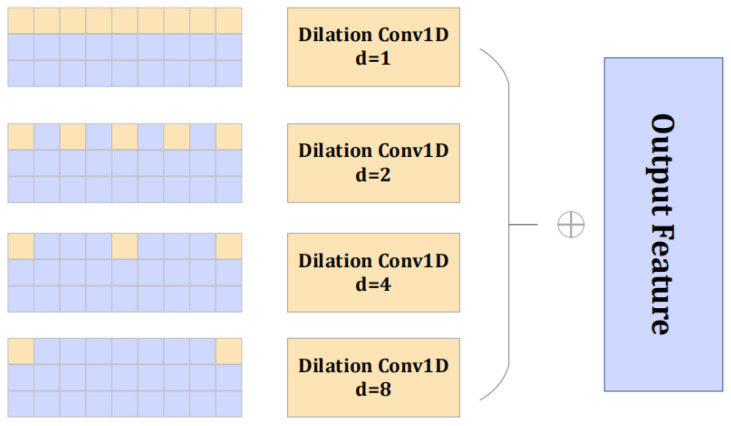
Multiscale temporal attention module.

The backbone X3D is pre-trained by Kinetics-400 (K400), a large-scale action recognition benchmark dataset. Meanwhile, non-local attention is adopted to process the feature maps obtained by the X3D. To ensure clarity and reproducibility, the framework is constructed from publicly available components: the X3D backbone [8] and the non-local attention module [9] are implemented according to their original publications. Our key contribution lies in the novel integration of these components with a dedicated multiscale temporal attention module and a tailored loss function, detailed in the following sections. These modifications are modular and can be implemented within standard deep-learning frameworks such as PyTorch (The version number is 1.13.0). However, in the anomaly detection task, the local correlation in the temporal dimension is also of indisputable importance. For this reason, a pyramid structure is introduced, and one-dimensional dilated convolution is used to deeply explore the multiscale characteristics of video segments in the time dimension. The multiscale temporal attention module helps learn multiscale feature from the pre-extracted features $F={{\left[fd\right]}^{D}}_{d=1}$. Given the features $f_{d}\in {\mathbb{R}}^{T}$, the kernel $W_{k,d}^{\left(l\right)}\in {\mathbb{R}}^{W}$ of the one-dimensional dilated convolution operator, where k∈{1, …, D/8}, d∈{1, …, D}, $l\in \{PDC_{1},\;PDC_{2},\;PDC_{3},\;PDC_{4}\}$. The multiscale temporal attention can be represented by Equation (1) and Figure 3.

(1) $$f_{k}^{\left(l\right)}=\sum_{d=1}^{D}{W_{k,d}^{\left(l\right)}}^{*(l)}f_{d}$$

where *(l) represents the index of the dilated convolution operator, $f_{k}^{\left(l\right)}\in {\mathbb{R}}^{T}$ represents the output feature after applying the dilated convolution in the temporal dimension, and the different dilated convolution operators with different are {1,2,4,8}.

#### 3.1.3. Top-K Sample Selection and Loss Function

After being enhanced by the nonlocal and multiscale temporal attention, these optimized feature vectors will pass through the classifier to generate scores corresponding to video frames. Furthermore, the top K scores with the highest absolute values are filtered out from the scores of normal and abnormal video frames, respectively, namely Top-K sample selection. Based on this, the amplitude score loss is calculated. Subsequently, according to the frame indices corresponding to the loss of these highest K scores, the corresponding feature vectors are extracted to calculate the loss of amplitude features of the prominent K features. By quantitative analysis of the difference amplitudes among these maximum frame-level scores, the optimal K value is selected. The experimental results show that when K = 3, the maximum score difference is significant, indicating that the model performs best under this value.

Based on Top-K selection, losses are calculated for the selected scores and the corresponding features. The prototype of the score loss function is the cross-entropy loss function, but the input is only the scores of the normal and abnormal video frames. The specific representation is shown in Equation (2).

(2) $$l_{f}=\sum_{x\in \Omega (X)}-y\log(f(x)+(1-y)\log(1-f(x))$$

where x represents the video frame, y represents the label of the video where the video frame is located, and f represents the model operation.

To increase the distinction of the loss function between normal and abnormal videos, in terms of features, Top-K selection is also used to select the features with the largest differences. Through the L2 norm, the features can be converted into a positive number, and this positive number can represent the score corresponding to the feature. The L2 norm of the scores of K video frames is calculated in the video, as shown in Equation (3). Next, the loss of feature amplitude is calculated by Equations (4) and (5), respectively.

(3) $$g\left(X\right)=\max_{\Omega_{k}(x)\subseteq {\left\{x_{i}\right\}}_{i=1}^{N}}\sum_{x_{t}\in \Omega_{k}(X)}∥f(x_{i}){∥}^{2}$$

(4) $$d(X^{+},X^{-})=g_{k}(X^{+})-g_{k}(X^{-})$$

(5) $$l_{s}=\max(0,m-d(X_{i},X_{j}))$$

where $X^{+}$ means a normal sample, while $X^{-}$ means an anomaly.

Normal and abnormal frames in the video often appear continuously. That is, several adjacent frames of a normal frame or an abnormal frame are also normal frames or abnormal frames, respectively. Therefore, the smoothness constraint loss (SCL) is added, so that after multiple trainings, the scores between adjacent normal frames and abnormal frames in a video are relatively close. Here, λ is a preset hyperparameter, which limits the value of the time-smoothing loss function within a reasonable range, and i represents the index of the current frame in the video, as shown in Equation (6).

(6) $$l_{c}=\lambda \sum_{i}^{\left(n-1\right)}(f(x_{i})-f(x_{i+1}){)}^{2}$$

Finally, the loss function consists of three parts, the amplitude score loss function, the amplitude feature loss function, and the time-smoothing loss function, as shown in Equation (7).

(7) $$\begin{array}{cc} l & =l_{f}+l_{s}+l_{c}\\ & =\sum_{x\in \Omega (X)}-y\log(f(x)+(1-y)\log(1-f(x))\\ & +\max(0,m-d(X_{i},X_{j}))\\ & +\lambda \sum_{i}^{\left(n-1\right)}(f(x_{i})-f(x_{i+1}){)}^{2} \end{array}$$

### 3.2. Track Anomaly Location Algorithm Based on Unsupervised Background Extraction

After detecting abnormal video frames, an unsupervised foreground segmentation method is introduced in this paper to accurately locate the position of foreign objects. This method does not rely on pixel-level annotations, making it suitable for scenarios where annotation resources are limited in practical engineering. Through the multi-scale VAE (Variational Autoencoder) structure and pixel-level background weight design, the model can still maintain high segmentation accuracy in complex background and small target scenarios.

**Figure 4:**
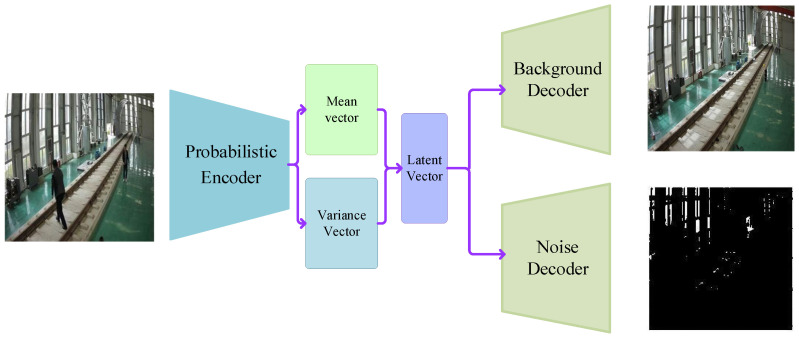
Architecture diagram of the VAE.

For a segmentation task, as shown in Figure 4, VAE first uses the decoder to analyse the input image and then generates the mean vector and the variance vector representing its internal information. Based on these statistical parameters, the model further synthesises a latent vector containing the input image information. This latent vector then passes through two different decoding paths. On the one hand, the background decoder focusses on analysing and reconstructing the background of the image. However, the noise decoder is dedicated to capturing and separating random noise in the input image.

**Figure 5:**
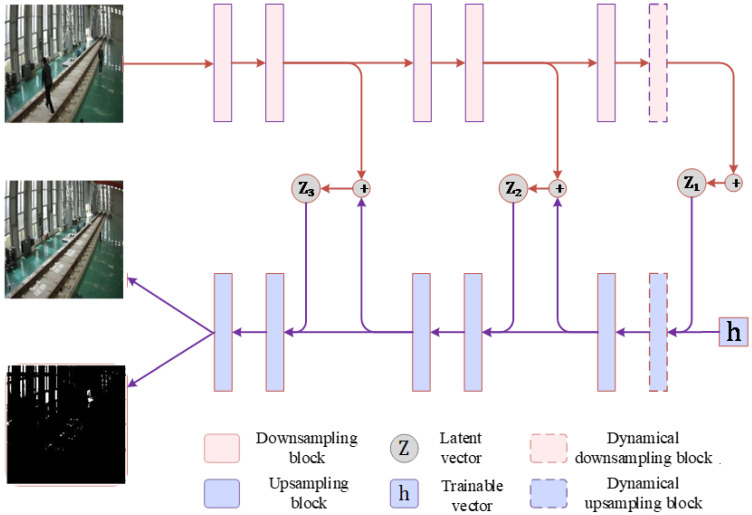
Multiscale encoder (decoder) architecture sharing parameters.

The basic VAE is highly sensitive to lighting, leading to misclassification of the foreground in areas with dramatic lighting changes, while potentially missing smaller foreign object details in poorly lit environments. Additionally, the model exhibits blurry performance in background generation, especially when the input image size increases, making it challenging to simultaneously enhance the clarity of the output background. Therefore, the unique feature of this model is that it divides the encoder and decoder modules into three progressive levels. In the first two levels, two layers of upsampling/downsampling modules are configured, respectively. After these layers, the low-dimensional variance and mean feature vectors are extracted, and then the latent representation space is constructed. The third level integrates a layer of conventional upsampling/downsampling modules and a layer of dynamical upsampling/downsampling modules. A dynamical layer is enabled as needed to generate higher-dimensional latent variables. These latent variables at different levels capture different scale image features, from fine- to coarse-grained. All of the above descriptions are illustrated in Figure 5.

By adopting this multi-level VAE architecture, the model effectively integrates the latent image feature information at various scales. It is worth noting that, due to parameter sharing, the parameters for three VAEs operating in parallel are equivalent to those of a single VAE with the highest complexity. This design enables efficient parameter reuse between the low-level and high-level VAE structures.

In the decoder part, the separated background image decoder and noise image decoder are merged into a unified encoder, which outputs four-channel features, with the aim of promoting in-depth fusion among different latent variables. In addition, in the double layer upsampling/downsampling modules of the third level, a layer of adaptively adjustable upsampling/downsampling layer is specially introduced, aiming to enhance the adaptability and processing flexibility of the model for input images of different sizes.

To enhance the adaptability and generalisability for input images of different sizes, the corresponding number of sampling layers is determined on the basis of the input images of different sizes. Specifically, for smaller images (for example, less than or equal to 500 × 500 pixels), 5 sampling blocks are selected and the number of convolutional channels is set to {3, 64, 160, 160, 32, 16}. For larger images, 6 sampling blocks are set and the corresponding number of convolutional channels is set to {3, 64, 160, 160, 160, 32, 16}. This allocation of the number of channels can ensure that the model fully captures the features of the image with a limited number of parameters, thereby achieving efficient and accurate feature extraction. It can not only maintain low computational complexity, but also dig and integrate image features at a deeper level, further improving the performance and robustness of the model in complex scenarios.

#### 3.2.1. Design of Encoder and Decoder Architecture

To locate the position of foreign objects more accurately, the spatial position encoding is added to two encoding channels, namely, the row encoding channel and the column encoding channel. In each convolutional block for encoding and deconvolutional block for decoding, it is expressed as follows.

(8) $$\left\{\begin{array}{c} Row\_encoding[i,j]=\frac{i}{h-1}-0.5\\ Column\_encoding[i,j]=\frac{j}{w-1}-0.5 \end{array}\right.$$

where h and w represent the encoding of the height and width of the image, respectively, i and j are the row index and column index, respectively.

**Figure 6:**
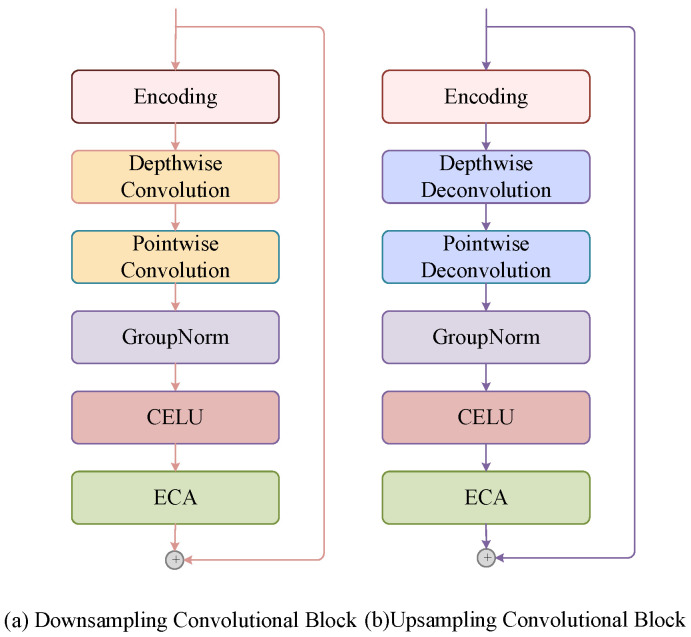
Downsampling convolutional block and upsampling convolutional block.

Traditional convolutional modules have a large computation and numerous parameters. Therefore, as shown in Figure 6, this paper proposes the use of depth-wise convolution and point-wise convolution to replace conventional convolution operations [56]. Then, the Group Normalisation layer and the Continuously Differentiable Exponential Linear Unit (CELU) activation function are used to reduce overfitting and improve the generalisation ability. To enhance the ability of the model to distinguish and focus on the features of each convolutional channel and strengthen its robustness to illumination noise, Efficient Channel Attention (ECA) [57] is introduced into the network. Through the application of this attention module, the model can explore multichannel information more meticulously, thereby improving the quality of depicting and reconstructing complex backgrounds.

#### 3.2.2. Pixel-by-Pixel Background Weight Coefficient

Compared to other image generation models, background generation places greater emphasis on the distinction between background and foreground information. To enhance the reconstruction loss, a background coefficient is introduced, which makes the reconstruction loss, which originally focused solely on image generation. It is more suitable for the background generation subtask. The calculation process is detailed in Equations (9)–(14).

(9) $$l_{n,i,j}=\sum_{c=1}^{3}\left|{\hat{x}}_{n,c,i,j}-x_{n,c,i,j}\right|$$

(10) $$m_{n,i,j}=\tanh(\frac{l_{n,i,j}}{\tau })$$

(11) $${\tilde{m}}_{n,i,j}(X,X_{n})=\frac{1}{{\left(2k+1\right)}^{2}}\sum_{l=-k,p=-k}^{l=k,p=-k}m_{n,i+1,j+p},\;k=[w/\gamma ]$$

(12) $$L_{rec}(\hat{X},X)=\frac{1}{Nhw}\sum_{n=1,i=1,j=1}^{N,h,w}e^{-\beta {\tilde{m}}_{n,i,j}}l_{n,i,j}$$

Here, $x_{n,c,i,j}$ is denoted as the value of the (i,j) pixel in the *c*-th channel of image $X_{n}$, where 1≤i≤h, 1≤j≤w, and h and w represent the height and width of the image, respectively. Similarly, ${\hat{x}}_{n,c,i,j}$ represents the pixel value of the reconstructed background at the same position. Then, the local reconstruction loss of pixel (i,j) based on the L1 norm can be described by Equation (9). After the above processing, the mask information is too blurred and incoherent. Therefore, a smoothing filtering process can be added to increase the smoothness of the mask. A square kernel with a size of (2k+1)×(2k+1) is used to calculate the average of the mask. Considering the continuity of foreground and background pixels, the nearby pixels of foreground pixels are probably foreground pixels. The nearby pixels of the background pixels are most likely the background pixels. Additionally, if the sizes of the input images are different, the number of pixels occupied by the foreground mask is also different. Therefore, k is set with w and γ, a positive hyperparameter that can adjust the threshold to distinguish between background and foreground, and [·] denotes the rounding function. Finally, the error caused by the foreground is amplified, making the reconstruction loss with a hyperparameter β tend to reconstruct the background rather than the entire image.

#### 3.2.3. Loss Function

As the weight $w_{n,i,j}$ changes, the reconstruction loss will change according to the different weight values of each pixel. For pixels considered background pixels, their weights are close to 1, and the reconstruction loss will be more affected by these pixels. For pixels that are not background pixels, their weights are close to 0, and the reconstruction loss is less affected by these pixels.

Therefore, in the VAE, the model adds a decoder with only one output channel, which is used to decode the estimation $l_{n,i,j}$ of the pixel error ${\hat{l}}_{n,i,j}$. The previously calculated background weight coefficient is added to it for weighting, so that the loss is mainly limited to the background area, as shown in Equation (13).

(13) $$L_{noise}=\frac{1}{3Nhw}\sum_{n=1,i=1,j=1}^{N,h,w}w_{n,i,j}[{\hat{l}}_{n,i,j}-l_{n,i,j}]$$

The multi-scale VAE architecture contains latent variables at multiple scales (levels). Each scale of latent variables has the corresponding means and variances, and they all affect the KL divergence loss. For a multiscale hierarchical VAE, the total KL divergence can be defined as follows.

(14) $$KL(q(z|x)∥p(z))=\sum_{l=1}^{L}E_{q(z_{<l}|x)}[KL(q(z_{l}|x,z_{<l})∥p(z_{l}|z_{<l}))]$$

where $z_{l}$ represents the latent variable at the l-th scale level. L is the total number of scale levels. $q(z_{l}|x,z<l)$ is the approximate posterior distribution of the high-level latent variable z given the observed data x and the low-level latent variable $z_{<l}$. $p(z_{l}|z_{<l})$ is the corresponding prior distribution.

Then the KL divergence can be represented by Equation (15).

(15) $$L_{KL}=\frac{1}{2}\sum_{i=1}^{d}\left(\sigma^{2}+\mu^{2}-\log\sigma^{2}-1\right)$$

(16) $$L=\lambda_{1}L_{rec}+\lambda_{2}L_{noise}+\lambda_{3}L_{KL}$$

where the mean of the latent variable generated by the encoder network is μ and the variance is $\sigma^{2}$. The final loss function is the weighted sum of the reconstruction loss based on the background weight, the noise estimation loss based on the background weight, and the KL regularization loss. As shown in Equation (16), $\lambda_{1}$, $\lambda_{2}$, and $\lambda_{3}$ are weight coefficients.

### 3.3. Dataset

The experiment is conducted using a real track at the experimental base. The camera is installed on one side of the track to detect foreign objects in the direction of a single train’s travel. The horizontal and vertical distances between the camera and the centre of the track line are both 2 m. The cameras used in the experimental base are Hikvision Smart265 cameras (Manufactured by Hangzhou Hikvision Digital Technology Co., Ltd., located in China.) with a focal length of 6 mm. The video resolution of the videos is 2540×1440 and the frame rate is 25 Frames Per Second (FPS).

Foreign objects in track intrusion typically include hard objects such as stones and concrete blocks, soft objects such as plastic sheets and kites, liquid substances such as debris flow and torrential flood, and living organisms such as humans and animals. In the experiment, some typical foreign objects are simulated, including persons, helmets, boxes of various sizes, bags, long steel, and ponding, etc. The track videos film in the Huanghua laboratory are sampled evenly at intervals of about 20 s to obtain video clips with a duration of 2 s. These video clips are classified according to the types of foreign objects, including four categories, normal, abnormal (human), abnormal (object), and abnormal (ponding). The data set is divided into a training set and a testing set in an 8:2 ratio. Table 2 shows the division of the training set and the testing set of the Huanghua track anomaly detection dataset.

**Figure 7:**
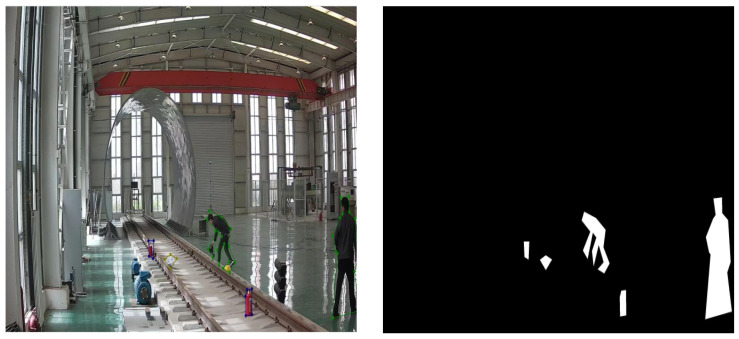
Target contour and foreground mask. The left subfigure shows the target contour image, while the right subfigure displays the foreground mask image.

LabelMe [58] can mark the standard contours of abnormal objects, pedestrians, or ponds, which are converted into mask images of the foreground. The number of images in the training set and the test set is shown in Table 3. The target contour and foreground mask are shown in Figure 7. In foreign object intrusion scenarios, the training set uses only normal samples for model training, with no need for abnormal samples. During testing, normal and abnormal samples are used. However, image samples containing foreign objects are difficult to collect, resulting in a scarcity of abnormal samples. Since only normal samples are used during training, the use of a large number of normal samples for testing would lead to artificially inflated test accuracy. Therefore, only a small number of normal samples and abnormal samples are used in the testing process.

### 3.4. Experimental Environment and Evaluation Metrics

All models are optimized using Adam with weight decay of 0.005 and momentum of 0.9. The learning rate is initialised at 0.001 and is decreased by a factor of 10 after 8 and 11 epochs, respectively. For the X3D, the X width is 1, the X-neck is 2.25, and the X-depth is 2.2. The τ, β, and γ are set to 0.25, 6, and 320, respectively. The deep learning experiments are all carried out on the A5000 and the Intel (R) Xeon (R) E5-2620 v3 @ 2. 40 GHz CPU. The operating system is Ubuntu 20.04 and the CUDA version is 12.0.

The area under the receiver operating characteristic curve (AUC) is used to evaluate the classification performance of the model. The calculation of the AUC is as follows. Suppose the number of positive samples is P, the number of negative samples is N, TPR represents the True Positive Rate, and FPR represents the False Positive Rate, then TPR and FPR can be expressed by Equation (17).

(17) $$TPR=\frac{TP}{TP+FN},\;FPR=\frac{FP}{FP+TN}$$

where TP is the number of True Positives, FN is the number of False Negatives, FP is the number of False Positives, and TN is the number of True Negatives. The AUC is the area under the ROC curve and can be calculated by Equation (18).

(18) $$AUC=\int_{0}^{1}TPR(FPR^{-1}(u))du$$

where $FPR^{-1}$ represents the inverse function of FPR and μ is a variable ranging from 0 to 1.

The pixel-level F1 score metric evaluates the similarity between the generated foreground mask and the ground-truth mask by measuring the alignment of foreground pixels. Combining precision and recall, it offers a comprehensive assessment of the quality of background generation.

Precision indicates the proportion of pixels that are correctly classified as the mask in the generated foreground mask. Recall indicates the proportion of pixels in the ground-truth foreground mask that are correctly classified as the foreground. The calculation is shown in Equation (19).

(19) $$\mathrm{Precision}=\frac{TP}{TP+FP},\;\mathrm{Recall}=\frac{TP}{TP+FN}$$

The F1 score is used as a comprehensive evaluation of precision and recall. The range of values of the F1 score is 0 to 1, and a higher value indicates a better degree of match between the generated background and the ground truth background. Calculating the F1 score is shown in Equation (20).

(20) $$F1=\frac{2\times \mathrm{Precison}\times \mathrm{Recall}}{\mathrm{Precison}+\mathrm{Recall}}$$

**Figure 8:**
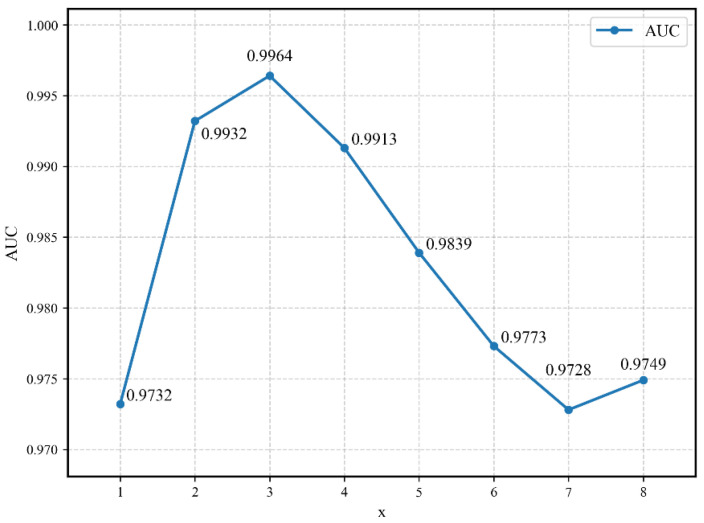
Plot of AUC under different values of the hyperparameter K.

In addition, based on the proposed model, eight groups of experiments are conducted for different values of K, and it is compared to determine whether the selection of the K value is related to the final AUC metric. Figure 8 shows that when *K* on the horizontal axis varies between 1 and 3, the AUC metric of the model increases as the value of *K* increases and when *K* on the horizontal axis varies between 3 and 8, the AUC metric of the model decreases as *K* increases. The highest AUC metric that can be obtained is 0.9964, and the lowest is 0.9732, indicating that the setting of the K parameter indeed has an impact on the final result and is sufficient to show that the proper *K* value can effectively improve the performance of the model.

## 4. Results

To verify the effectiveness of the improved algorithm for detecting foreign objects in videos, the impacts of three improvement modules on the performance of the video anomaly detection model, namely video preprocessing strategies, attention enhancement, and loss function optimisation, are systematically explored through ablation experiments, as shown in Table 4. Compared to baseline, preprocessing, the attention mechanism and the loss function improve the AUC metrics by 7.29%, 2.23% and 7.95%, respectively. Amplification of the far-point information and the mask can both improve the AUC of the model, which fully demonstrates the importance of constructing the track clearance as the ROI and processing the far-point information when dealing with railway scenes. The Top-K selection is implemented for baseline X3D, and the corresponding amplitude score/feature loss function is introduced, leading to a 7.01% increase in the AUC metric. It turns out that the effectiveness of the top-K and loss function optimisation in improving the discriminative ability of the model. More detailed information can be found in Table 4. The results demonstrate that the optimisation measures in these three aspects could all significantly improve the accuracy of the model, specifically reflected in the increase in AUC.

**Figure 9:**
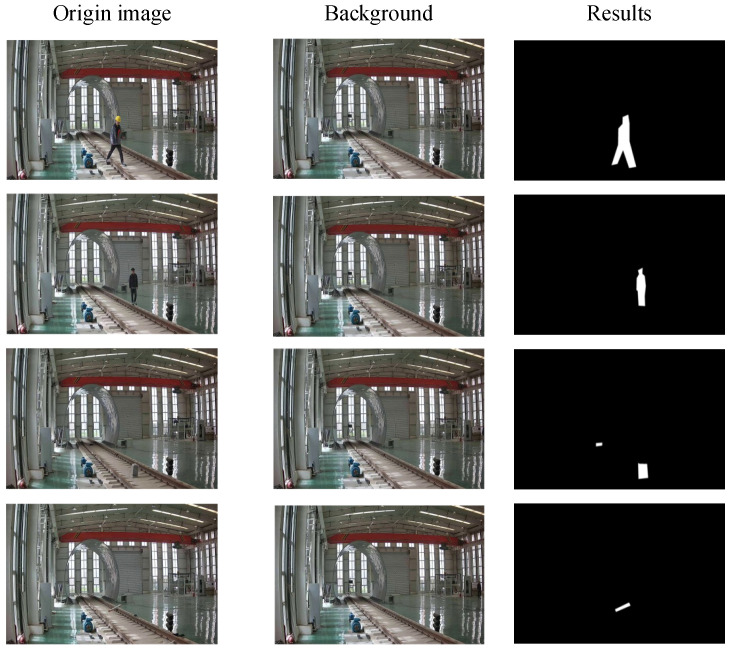
Output of algorithms in the Huanghua Railway anomaly location dataset. The first column presents the original images without any processing. The second column shows the background images obtained after the foreground is removed through the model. The third column presents images generated by the method in this paper.

To further obtain the location of foreign objects, anomaly localisation experiments are conducted on the Huanghua rail anomaly localisation data set using an unsupervised foreground segmentation algorithm. In this experiment, the algorithm is compared and analysed in detail with the basic VAE, AE-NE. Upon examining the data in Table 5, it is evident that the proposed algorithm shows better performance compared to other models in most of the evaluation metrics. Specifically, without considering the setting of ROI area and image cropping operations, the algorithm achieves improvements of 3.34%, 2.80%, and 3.08%, respectively, in the three key metrics of precision, recall, and F1 score. After incorporating ROI area setting and image cropping strategies, compared to baseline, the improvement ranges of the method on the above three metrics are further refined to 2.54%, 3.46%, and 3.03%. This result fully proves the effectiveness and superiority of the proposed algorithm. By observing Figure 9, in the typical sample cases shown in the third row, the baseline method fails to effectively identify the details of tiny foreign objects under faint shadows. However, the proposed algorithm detects and highlights them successfully. Therefore, it can be concluded that, compared to the baseline, the segmentation algorithm demonstrates a significant improvement in recognition performance when dealing with low-light environments and scenarios to identify tiny objects.

To better demonstrate the superiority of the proposed unsupervised foreground segmentation algorithm, Table 6 provides a comparison of the F1 scores between the proposed algorithm and other current advanced models in the CDnet2014 dataset. It is evident that, in terms of overall average performance, the model proposed in this paper has achieved an improvement of 5.34% compared to the second-best performing model, the Autoencoder with Noise Contrastive Estimation (AE-NE) [41]. Furthermore, our model has achieved the best performance in the categories of Bad Weather, Camera Jitter, Dynamic Background, Intermittent Object Motion, and Thermal. Specifically, compared to AE-NE, our model has achieved improvements of 5.76%, 3.67%, 26.78%, 0.15%, and 11.95% in these categories, respectively.

**Figure 10:**
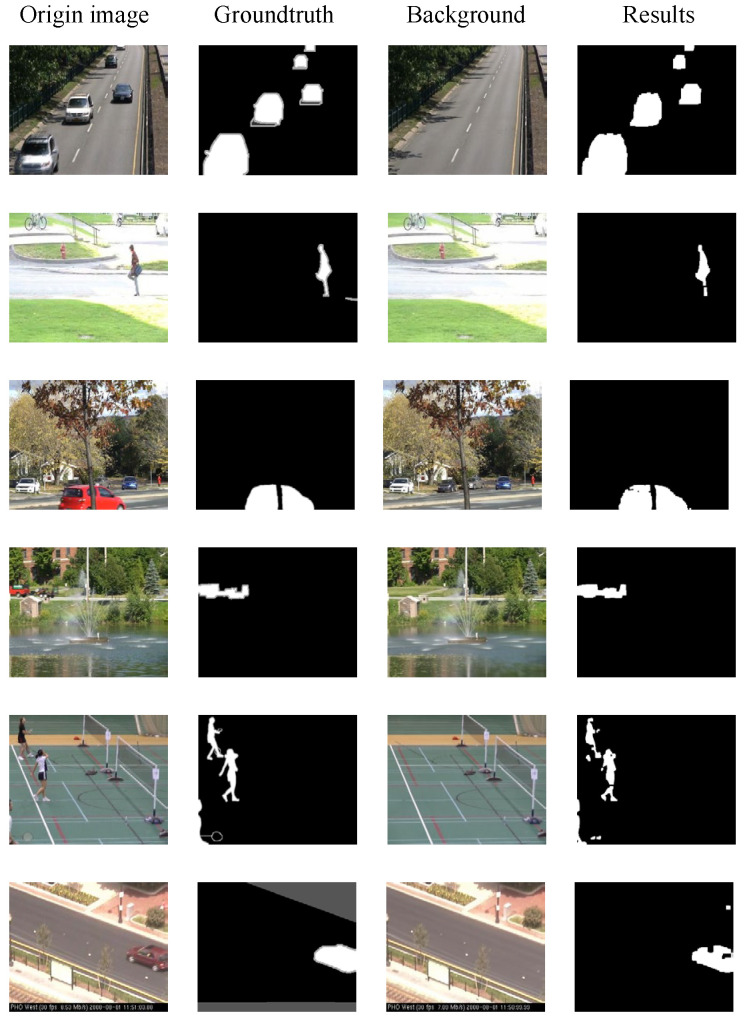
Outputting samples of the algorithm on the CDnet2014 dataset.

These results highlight the sound performance of the unsupervised foreground segmentation algorithm in a variety of complex environments, as shown in Figure 10. The algorithm is applicable to scenarios such as low illumination, heat flow disturbances, low frame rates, and other basic situations.

## 5. Discussion

With the increasing speed of trains and the growing number of risk factors, video-based monitoring for foreign object intrusion, which operates under all weather conditions, plays a crucial role in ensuring the safe operation of railways. To address data imbalances and the labour-intensive nature of data annotation in railway scenarios, a weakly supervised video anomaly detection method is proposed. It uses track clearance with ROI masks, far-point amplification, and global scene analysis for video preprocessing. Enhanced feature extraction is achieved using X3D with nonlocal and multiscale temporal attention mechanisms. The model is optimized through Top-K selection and tailored loss functions. After obtaining specific abnormal video frames, an unsupervised VAE-based segmentation algorithm further refines anomaly location by integrating spatial position encoding and optimising the sampling module to balance accuracy and efficiency. Comprehensive experiments carried out on relevant datasets demonstrate the superior generalisability and effectiveness of the proposed approach in detecting foreign object intrusions. Upon successful detection and segmentation of an intrusion, the system triggers real-time audiovisual alarms at the railway operation control centre. More critically, this alert can be directly integrated with the signalling system to automatically prompt approaching trains to slow down or initiate an emergency stop, thereby preventing potential collisions and ensuring the safety of passengers and infrastructure. This enables proactive measures such as alerting approaching trains to slow down or stop, thereby preventing potential collisions and ensuring the safety of passengers and infrastructure, while minimizing service disruptions. Additionally, the method proposed in this paper not only meets the requirements for high-speed rail wheelsets, but can also be applied in various scenarios that require video-based intrusion detection, such as maglev railways, highways, and airports.

Despite promising results, the proposed framework has certain limitations. In terms of processing speed, while the system processes a 2 s clip in 0.41 s, achieving an end-to-end latency low enough for instantaneous braking decisions at the highest train speeds (e.g., over 300 km/h) remains a challenge, as the cumulative delay from detection to alarm dissemination and driver reaction must be critically minimized. Regarding potential hazards, the system is primarily designed for intrusion detection rather than risk assessment; it cannot distinguish between a high-risk obstacle (e.g., a large metal object) and a lower-risk one (e.g., a plastic bag), which could lead to unnecessary emergency braking if not coupled with a risk assessment module. Furthermore, the reliance on technical solutions based purely on visual data makes the system susceptible to performance degradation under extreme weather conditions (e.g., heavy fog or torrential rain) that severely obscure camera visibility, and it does not leverage complementary sensors such as LiDAR or radar for redundancy in such scenarios.

Although the detection of foreign objects is addressed in this paper, the specific location and size of these objects still need to be further estimated. Therefore, in future work, video foreign object intrusion detection under multi-scenario conditions will be further considered, particularly focusing on the robustness of the model under complex scenario conditions. By combining binocular vision and camera self-calibration, we aim to effectively estimate and evaluate the size and position of foreign objects, thus assessing the risk degree of foreign object intrusion on the track.

## Figures and Tables

**Table 1:** Comparison of different categories of research.

Category	Specific Method	Advantages	Disadvantages/Challenges
Traditional Vision Methods	[11,12,13]	Simple implementation, high computational efficiency, good real-time performance.	Sensitive to environmental changes, high false alarm rate; threshold selection significantly impacts accuracy.
Deep Learning-Based Methods (2D Images) (Supervised)	[16,22,24,25,26,27]	High accuracy/simple implementation	Requires large amounts of precisely annotated data/insufficient generalization to unseen object types
Deep Learning-Based Methods (3D Vision) (Supervised)	[15,28,29,30]	3-D location	Relies on expensive sensors/computationally-complex processing
Video Anomaly Detection (Unsupervised)	[33,34,35]	Low labelled cost	Requires highly accurate learning of normal patterns
Video Anomaly Detection (Weakly Supervised)	[23,36]		performance depends on the quality of video-level labels
Video Anomaly Detection (Self-Supervised)	[22,37]		depends on the design of the pretext task
Foreground Segmentation (Traditional Methods)	[49,50,51]	Low computational resource requirements	Performance degrades significantly under drastic illumination changes and complex dynamic backgrounds
Foreground Segmentation (Deep Learning Methods)	[52,53,54,55]	High robustness	Requires large amounts of training data
The Proposed Method	ALL	Low labelled cost/high robustness	High-precision algorithm design is required.

**Table 2:** Division of training and testing sets in the Huanghua track anomaly detection dataset.

Category	Train Set	Test Set	ALL
Normal video	176	44	220
Abnormal (person)	69	17	240
Abnormal (object)	83	21	
Abnormal (ponding)	40	10	
ALL	368	92	460

**Table 3:** Division of training and testing sets in the Huanghua track anomaly localization dataset.

Dataset	Number
Train set	4463
Test Set	40 (normal)
	70 (Abnormal)

**Table 4:** Video anomaly detection conducts ablation experiments.

Cate	Model	The Origin Size	The Input Size	Frames	AUC	Time (GPU)	Time (CPU)
Baseline	X3D-pre-train	2560 × 1440	512 × 288	4	87.12%	0.32 s	0.72 s
preprocessing	+Mask	1790 × 660	512 × 288	4	91.43%	0.27 s	0.56 s
	+Masked +Far-point Information Amplification	1790 × 660 × 2	512 × 189 × 2	8	94.41%	0.36 s	0.63 s
Attention	+Nonlocal	2560 × 1440	512 × 288	4	88.47%	0.37 s	0.79 s
	+MSTA	2560 × 1440	512 × 288	4	89.12%	0.35 s	0.67 s
	+Non-local + MSTA	2560 × 1440	512 × 288	4	89.35%	0.39 s	0.81 s
Loss function	+Amplitude Loss	2560 × 1440	512 × 288	4	94.13%	0.33 s	0.70 s
	+SCL	2560 × 1440	512 × 288	4	91.72%	0.32 s	0.71 s
	+Amplitude Loss + SCL	2560 × 1440	512 × 288	4	95.07%	0.37 s	0.74 s
Ours	ALL	1790 × 660 × 2	512 × 189 × 2	8	99.64%	0.41 s	0.87 s

**Table 5:** Experimental results in Huanghua rail anomaly localization dataset.

Method	Origin Size	Input Size	F1	Precision	Recall
VAE	2560 × 1440	640 × 320	0.7363	0.6601	0.8327
AE-NE	2560 × 1440	640 × 320	0.7852	0.8371	0.7526
Baseline	2560 × 1440	640 × 320	0.8174	0.8212	0.8139
Baseline + ROI	1790 × 660	600 × 220	0.8730	0.8838	0.8627
Ours	2560 × 1440	640 × 320	0.8482	0.8546	0.8419
Ours + ROI	1790 × 660	600 × 220	**0.9033** (+0.0859)	**0.9092** (+0.88)	**0.8973** (+0.834)

The bold black font represents the best performance.

**Table 6:** The experimental results of the proposed algorithm compared with other models on CDnet2014.

Methods	Ours	AE-NE [7]	IUTIS-5 [8]	WisenetMD [9]	SuBSENSE [10]
Baseline	0.8892	0.8959	**0.9567**	0.9487	0.9503
Bad.Wea.	**0.8913**	0.8337	0.8248	0.8616	0.8619
Cam.Jitt.	**0.9597**	0.9230	0.8332	0.8228	0.8152
Dyn.Back	**0.8903**	0.6225	0.8902	0.8376	0.8177
Inter.Obje.	**0.8246**	0.8231	0.7296	0.7264	0.6569
LowFrame.	**0.7888**	0.6771	0.7743	0.6404	0.6445
Night	0.5496	0.5172	0.5290	**0.5701**	0.5599
PTZ	**0.8367**	0.8000	0.4282	0.3367	0.3476
Shadow	0.8908	0.8947	**0.9084**	0.8984	0.8986
Thermal	**0.9194**	0.7999	0.8303	0.8152	0.8171
Turbu.	0.8305	**0.8382**	0.7836	0.8304	0.7792
Overall	**0.8375**	0.7841	0.7717	0.7535	0.7408

The bold black font represents the best performance, and the underlined text represents the second-best-performing model.

## Data Availability

The data that support the findings of this study are available from the corresponding author upon reasonable request.

## Abbreviations

The following abbreviations are used in this manuscript:

AUC	Area Under the receiver operating characteristic Curve
CNN	Convolutional Neural Network
ECA	Efficient Channel Attention
F1	F1 Score
FPS	Frames Per Second
KL	Kullback-Leibler divergence
ROI	Region Of Interest
SCL	Smoothness Constraint Loss
VAE	Variational Autoencoder
X3D	Xception 3D

## References

[1] Meng H. (2022). A Study of Intelligent Monitoring and Identification Methods for Rail Safety Based on Optical Fibre Sensing Technology. Ph.D. Dissertation.

[2] Guan L. (2019). A Study on Real-Time Sensing Technology for UAV-Based Railway Transportation Line Environments. Master’s Thesis.

[3] Deng X. (2022). A study on Unsupervised Methods for Foreign Object Detection on Railway Tracks. Master’s Thesis.

[4] Oh K., Yoo M., Jin N., Ko J., Seo J., Joo H., Ko M. (2022). A Review of Deep Learning Applications for Railway Safety. Appl. Sci..

[5] Tang Q., Wei X., Wei D., Shen X., Yin X., Wang D., Jia L., Zhong Q. (2024). High Precision Robust Real-Time Lightweight Approach for Railway Pantograph Slider Wear Estimation. IEEE Trans. Intell. Transport. Syst..

[6] Kaur H., Pannu H., Malhi A. (2019). A Systematic Review on Imbalanced Data Challenges in Machine Learning: Applications and Solutions. ACM Comput. Surv..

[7] Zhou Z.-H. (2018). A brief introduction to weakly supervised learning. Natl. Sci. Rev..

[8] Feichtenhofer C. X3D: Expanding Architectures for Efficient Video Recognition. Proceedings of the IEEE/CVF Conference on Computer Vision and Pattern Recognition.

[9] Wang X., Girshick R., Gupta A., He K. Non-local Neural Networks. Proceedings of the IEEE/CVF Conference on Computer Vision and Pattern Recognition.

[10] Kingma D.P., Welling M. (2022). Auto-Encoding Variational Bayes. arXiv.

[11] Oh S., Park S., Lee C. A platform surveillance monitoring system using image processing for passenger safety in railway station. Proceedings of the International Conference on Control, Automation and Systems.

[12] Li C., Xie Z., Qin Y., Jia L., Chen Q. (2021). A multiscale image and dynamic candidate region-based automatic detection of foreign targets intruding the railway perimeter. Measurement.

[13] Nguyen T.B., Nguyen V.T., Chung S.-T. (2015). A Real-time Pedestrian Detection based on AGMM and HOG for Embedded Surveillance. J. Korea Multimed. Soc..

[14] Meng C., Wang Z., Shi L., Gao Y., Tao Y., Wei L. (2023). SDRC-YOLO: A Novel Foreign Object Intrusion Detection Algorithm in Railway Scenarios. Electronics.

[15] Yanwei J., Yu D. Research on Railway Obstacle Detection Method Based on Radar. Proceedings of the 7th International Symposium on Mechatronics and Industrial Informatics.

[16] Ding X., Cai X., Zhang Z., Liu W., Song W. Railway Foreign Object Intrusion Detection based on Deep Learning. Proceedings of the International Conference on Computer Engineering and Artificial Intelligence.

[17] Chen W., Meng S., Jiang Y. (2022). Foreign Object Detection in Railway Images Based on an Efficient Two-Stage Convolutional Neural Network. Comput. Intell. Neurosci..

[18] Ponimatkin G., Samet N., Xiao Y., Du Y., Marlet R., Lepetit V. (2022). A Simple and Powerful Global Optimization for Unsupervised Video Object Segmentation. arXiv.

[19] Yang Z., Wang Q., Bertinetto L., Hu W., Bai S., Torr P.H.S. (2019). Anchor Diffusion for Unsupervised Video Object Segmentation. arXiv.

[20] Zhang Y., Li L., Wang W., Xie R., Song L., Zhang W. (2023). Boosting Video Object Segmentation via Space-time Correspondence Learning. arXiv.

[21] Tokmakov P., Li J., Gaidon A. (2023). Breaking the “Object” in Video Object Segmentation. arXiv.

[22] Zhang Y., Song J., Jiang Y., Li H. (2023). Online Video Anomaly Detection. Sensors.

[23] Zhang Y.J. (1996). A survey on evaluation methods for image segmentation. Pattern Recognit..

[24] Jiang P., Ergu D., Liu F., Cai Y., Ma B. (2022). A Review of Yolo Algorithm Developments. Procedia Comput. Sci..

[25] Tian Y., Pang G., Chen Y., Singh R., Verjans J.W., Carneiro G. (2021). Weakly Supervised Video Anomaly Detection with Robust Temporal Feature Magnitude Learning. arXiv.

[26] Wang G., Wang Y., Qin J., Zhang D., Bao X., Huang D. (2022). Video Anomaly Detection by Solving Decoupled Spatiotemporal Jigsaw Puzzles. arXiv.

[27] Purwanto D., Chen Y.-T., Fang W.-H. Dance with Self-Attention: A New Look of Conditional Random Fields on Anomaly Detection in Videos. Proceedings of the IEEE/CVF International Conference on Computer Vision.

[28] Charles R.Q., Su H., Kaichun M., Guibas L.J. PointNet: Deep Learning on Point Sets for 3D Classification and Segmentation. Proceedings of the IEEE Conference on Computer Vision and Pattern Recognition.

[29] Shi S., Wang X., Li H. (2019). PointRCNN: 3D Object Proposal Generation and Detection from Point Cloud. arXiv.

[30] Qi S., Yu D. (2021). Railway obstacle detection based on radar and image data fusion. J. Phys. Conf. Ser..

[31] Hussain M., O’Nils M., Lundgren J., Mousavirad S.J. (2024). A Comprehensive Review on Deep Learning-Based Data Fusion. IEEE Access.

[32] Zhou H., Yu J., Yang W. (2023). Dual Memory Units with Uncertainty Regulation for Weakly Supervised Video Anomaly Detection. arXiv.

[33] Zaheer M.Z., Mahmood A., Khan M.H., Segu M., Yu F., Lee S.-I. (2022). Generative Cooperative Learning for Unsupervised Video Anomaly Detection. arXiv.

[34] Cho M., Kim T., Kim W.J., Cho S., Lee S. (2022). Unsupervised Video Anomaly Detection via Normalizing Flows with Implicit Latent Features. arXiv.

[35] Sun C., Jia Y., Song H., Wu Y. (2021). Adversarial 3D Convolutional Auto-Encoder for Abnormal Event Detection in Videos. IEEE Trans. Multimed..

[36] Li G., Cai G., Zeng X., Zhao R., Avidan S., Brostow G., Cissé M., Farinella G.M., Hassner T. (2022). Scale-Aware Spatio-temporal Relation Learning for Video Anomaly Detection. Proceedings of the Computer Vision—ECCV 2022.

[37] Wu J.-C., Hsieh H.-Y., Chen D.-J., Fuh C.-S., Liu T.-L., Avidan S., Brostow G., Cissé M., Farinella G.M., Hassner T. (2022). Self-supervised Sparse Representation for Video Anomaly Detection. Proceedings of the Computer Vision—ECCV 2022.

[38] Garcia-Garcia B., Bouwmans T., Rosales Silva A.J. (2020). Background subtraction in real applications: Challenges, current models and future directions. Comput. Sci. Rev..

[39] Bouwmans T. (2014). Traditional and recent approaches in background modeling for foreground detection: An overview. Comput. Sci. Rev..

[40] An Y., Zhao X., Yu T., Guo H., Zhao C., Tang M., Wang J. (2023). ZBS: Zero-shot Background Subtraction via Instance-level Background Modeling and Foreground Selection. arXiv.

[41] Sauvalle B., De La Fortelle A. Autoencoder-based background reconstruction and foreground segmentation with background noise estimation. Proceedings of the IEEE/CVF Winter Conference on Applications of Computer Vision.

[42] Sauvalle B., de La Fortelle A. (2022). Fast and Accurate Background Reconstruction Using Background Bootstrapping. J. Imaging.

[43] Ding H., Liu C., He S., Jiang X., Torr P.H.S., Bai S. (2023). MOSE: A New Dataset for Video Object Segmentation in Complex Scenes. arXiv.

[44] Xi L., Chen W., Wu X., Liu Z., Li Z. (2024). Online Unsupervised Video Object Segmentation via Contrastive Motion Clustering. IEEE Trans. Circuits Syst. Video Technol..

[45] Ren S., Liu W., Liu Y., Chen H., Han G., He S. Reciprocal Transformations for Unsupervised Video Object Segmentation. Proceedings of the IEEE/CVF Conference on Computer Vision and Pattern Recognition.

[46] Lu X., Wang W., Ma C., Shen J., Shao L., Porikli F. (2020). See More, Know More: Unsupervised Video Object Segmentation with Co-Attention Siamese Networks. arXiv.

[47] Cho S., Lee M., Lee S., Park C., Kim D., Lee S. (2022). Treating Motion as Option to Reduce Motion Dependency in Unsupervised Video Object Segmentation. arXiv.

[48] Lee S., Cho S., Lee D., Lee M., Lee S. Tsanet: Temporal and Scale Alignment for Unsupervised Video Object Segmentation. Proceedings of the IEEE International Conference on Image Processing.

[49] Barnich O., Van Droogenbroeck M. (2011). ViBe: A Universal Background Subtraction Algorithm for Video Sequences. IEEE Trans. Image Process..

[50] St-Charles P.-L., Bilodeau G.-A., Bergevin R. A Self-Adjusting Approach to Change Detection Based on Background Word Consensus. Proceedings of the IEEE Winter Conference on Applications of Computer Vision.

[51] St-Charles P.-L., Bilodeau G.-A., Bergevin R. (2015). SuBSENSE: A universal change detection method with local adaptive sensitivity. IEEE Trans Image Process.

[52] Lim L.A., Yalim Keles H. (2018). Foreground segmentation using convolutional neural networks for multiscale feature encoding. Pattern Recognit. Lett..

[53] Tezcan M.O., Ishwar P., Konrad J. (2021). BSUV-Net 2.0: Spatiotemporal Data Augmentations for Video-Agnostic Supervised Background Subtraction. IEEE Access.

[54] Yang Y., Ruan J., Zhang Y., Cheng X., Zhang Z., Xie G. (2022). STPNet: A Spatial-Temporal Propagation Network for Background Subtraction. IEEE Trans. Circuits Syst. Video Technol..

[55] Akilan T., Wu Q.J., Safaei A., Huo J., Yang Y. (2020). A 3D CNN-LSTM-Based Image-to-Image Foreground Segmentation. IEEE Trans. Intell. Transport. Syst..

[56] Barron J.T. (2017). Continuously Differentiable Exponential Linear Units. arXiv.

[57] Wang Q., Wu B., Zhu P., Li P., Zuo W., Hu Q. ECA-Net: Efficient Channel Attention for Deep Convolutional Neural Networks. Proceedings of the IEEE/CVF Conference on Computer Vision and Pattern Recognition.

[58] Russell B.C., Torralba A., Murphy K.P., Freeman W.T. (2008). LabelMe: A Database and Web-Based Tool for Image Annotation. Int. J. Comput. Vis..

